# *Frataxin* is essential for zebrafish embryogenesis and pronephros formation

**DOI:** 10.3389/fcell.2024.1496244

**Published:** 2024-12-11

**Authors:** Wesley S. Ercanbrack, Austin Dungan, Ella Gaul, Mateo Ramirez, Alexander J. DelVecchio, Calvin Grass, Rebecca A. Wingert

**Affiliations:** Department of Biological Sciences, University of Notre Dame, Notre Dame, IN, United States

**Keywords:** frataxin, kidney, nephron, metabolism, development, zebrafish

## Abstract

**Background and objectives:**

Friedreich’s Ataxia (FRDA) is a genetic disease that affects a variety of different tissues. The disease is caused by a mutation in the *frataxin* gene (*FXN)* which is important for the synthesis of iron-sulfur clusters. The primary pathologies of FRDA are loss of motor control and cardiomyopathy. These occur due to the accumulation of reactive oxygen species (ROS) in the brain and the heart due to their high metabolic rates. Our research aims to understand how developmental processes and the kidney are impacted by a deficiency of *FXN*.

**Methods:**

We utilized an antisense oligomer, or morpholino, to knockdown the *frataxin* gene (*fxn*) in zebrafish embryos. Knockdown was confirmed via RT-PCR, gel electrophoresis, and Sanger sequencing. To investigate phenotypes, we utilized several staining techniques including whole mount *in situ* hybridization, Alcian blue, and acridine orange, as well as dextran-FITC clearance assays.

**Results:**

*fxn* deficient animals displayed otolith malformations, edema, and reduced survival. Alcian blue staining revealed craniofacial defects in *fxn* deficient animals, and gene expression studies showed that the pronephros, or embryonic kidney, had several morphological defects. We investigated the function of the pronephros through clearance assays and found that the renal function is disrupted in *fxn* deficient animals in addition to proximal tubule endocytosis. Utilizing acridine orange staining, we found that cell death is a partial contributor to these phenotypes.

**Discussion and conclusion:**

This work provides new insights about how *fxn* deficiency impacts development and kidney morphogenesis. Additionally, this work establishes an additional model system to study FRDA.

## 1 Introduction

Friedreich’s Ataxia (FRDA, OMIM #229300) is an autosomal recessive neurodegenerative disease and the most common inheritable ataxia (Anheim et al., 2010; Anheim et al., 2012). FRDA is caused by genetic mutations that reduce expression of the protein Frataxin (FXN). The vast majority (96%–98%) of patients have variable numbers of trinucleotide GAA repeats in the first intron of the *FXN* gene, though genetic lesions encoding other mutations have also been identified (Reetz et al., 2018; Gottesfeld, 2019). Patients typically begin losing motor function between 5 and 15 years of age (Delatycki et al., 1999; Dürr et al., 1996; Filla et al., 1990; Parkinson et al., 2013; Schöls et al., 1997), which is thought to be due to cerebellar and dorsal root ganglia degeneration (Koeppen et al., 2011; Koeppen and Mazurkiewicz, 2013). Other neurological symptoms include dysarthria, oculomotor abnormalities and hearing loss (Fahey et al., 2008; Schöls et al., 1997). While the previously mentioned symptoms are primarily neurological, there are many FRDA symptoms that are not. Scoliosis, foot deformities, and cardiomyopathy are the most common, non-ataxia symptoms of FRDA (Reetz et al., 2018). Interestingly, each of these phenotypes are positively correlated in severity and occurrence with the number of GAA repeat expansions in intron 1 of *FXN* (Filla et al., 1996; Reetz et al., 2018).

FXN is a mitochondrial protein involved in the biosynthesis of iron-sulfur clusters (ISC) (Adinolfi et al., 2002; Adinolfi et al., 2009; Colin et al., 2013; Huynen et al., 2001; Mühlenhoff et al., 2002; Tsai and Barondeau, 2010; Ventura et al., 2006). ISCs are used to make proteins for the electron transport chain, citric acid cycle, fatty acid biosynthesis, and glycolysis (Maio and Rouault, 2022). For example, ISCs are critical cofactors for aconitase and electron transport chain complexes I, II, and III. Decreased FXN levels lessen antioxidant defenses and the accrual of unused iron generates free radicals. Together, these changes cause cells to accumulate oxidative damage (Pallardó et al., 2021; Sivakumar and Cherqui, 2022; Kelekçi et al., 2022). Cell death is thought to transpire from this damage and insufficient energy (ATP) production (Pallardó et al., 2021; Sivakumar and Cherqui, 2022; Kelekçi et al., 2022). Tissues with high metabolic demands that are rich in mitochondria, like neurons and myocardium, are disproportionately affected over time (Koutnikova et al., 1997). However, other organs with high metabolic demands, like the kidney (Elia, 1992), are seemingly unaffected in FRDA. This is quite surprising, as related ataxias and mitochondrial disorders are very frequently characterized by renal diseases, such as the nephrotic syndrome (Martín-Hernández et al., 2005; Diomedi-Camassei et al., 2007; Quinzii and Hirano, 2010; Emma et al., 2012; Ashraf et al., 2013). As such, much still remains to be learned about how FXN deficiency affects many tissues, organs, and systems across the body.

Researchers have utilized several different model organisms to study the biological functions of FXN. Bacteria, yeast, plants, worms, and flies have allowed for important discoveries about its roles (Busi et al., 2006; Kelekçi et al., 2022; Mühlenhoff et al., 2002), as well as knockdown mouse models, knockdown mouse fibroblast cell cultures, and FRDA patient cell cultures (Kelekçi et al., 2022; Perdomini et al., 2013). In particular, murine models include a useful array of various partial or conditional *Fxn* knockdown strategies, the latter including tissue-specific models which enable analysis of the effects of FXN-deficiency on particular cell types. Complete knockout of *Fxn* is embryonic lethal in mice (Cossée et al., 2000), which has precluded assessment of the early developmental roles for FXN in mammals. Mouse fibroblast *Fxn* deletions also lead to cell death (Calmels et al., 2009). These studies coincide with the observation that no FRDA patients have been reported to be homozygous with deleterious point mutations which would result in a complete absence of FXN (Perdomini et al., 2013).

In the present study, we utilized the zebrafish, *Danio rerio*, to study the consequence of severe *fxn* loss of function during ontogeny, as this animal model provides unique opportunities to examine the events of early embryogenesis. Zebrafish develop externally in clear chorions which allows for direct observation of vertebrate development beginning at fertilization. Further, the zebrafish genome is highly conserved with humans (Howe et al., 2013) and shares many of the genetic regulatory mechanisms that govern development, as well as many physiological and cellular processes.

Here, we designed a knockdown zebrafish model to specifically target and reduce zygotic *fxn* expression while preserving the expression of maternal *fxn* transcripts, thus enabling development in the context of attenuated Fxn levels. In this model, we found that *fxn-*deficient zebrafish exhibited poor growth and failure to thrive, with compromised survival. Additionally, *fxn-*deficient zebrafish embryos exhibited morphological defects across numerous tissues including the central nervous system, otoliths, craniofacial structures, and the pronephros, or embryonic kidney. Within the kidney, multiple lineages were reduced, which was associated with elevated cell death and correlated with compromised ability to clear fluid and maintain water homeostasis, leading to edema. These findings illustrate that *fxn* plays roles in the formation of the kidney, and this new loss of function model provides an opportunity to further delineate the roles of *fxn* during vertebrate development.

## 2 Results

### 2.1 Molecular and computational validation of *Fxn* deficiency

A complete *Fxn* knockout in mice is embryonic lethal (Cossée et al., 2000). As a result, several conditional *Fxn* knockdown mouse models have been developed (Kelekçi et al., 2022). Studies using these conditional mouse models have suggested that *Fxn* may have roles in development (Chandran et al., 2017; Jiralerspong et al., 1997; Koeppen et al., 2017; Santos et al., 2001). These mouse models as well as work in systems ranging from bacterial to FRDA patient derived cell lines have contributed greatly to our understanding of FRDA pathology, but they have a limited ability to answer the question of how FXN deficiency impacts early aspects of embryonic development such as organogenesis.

**FIGURE 1:**
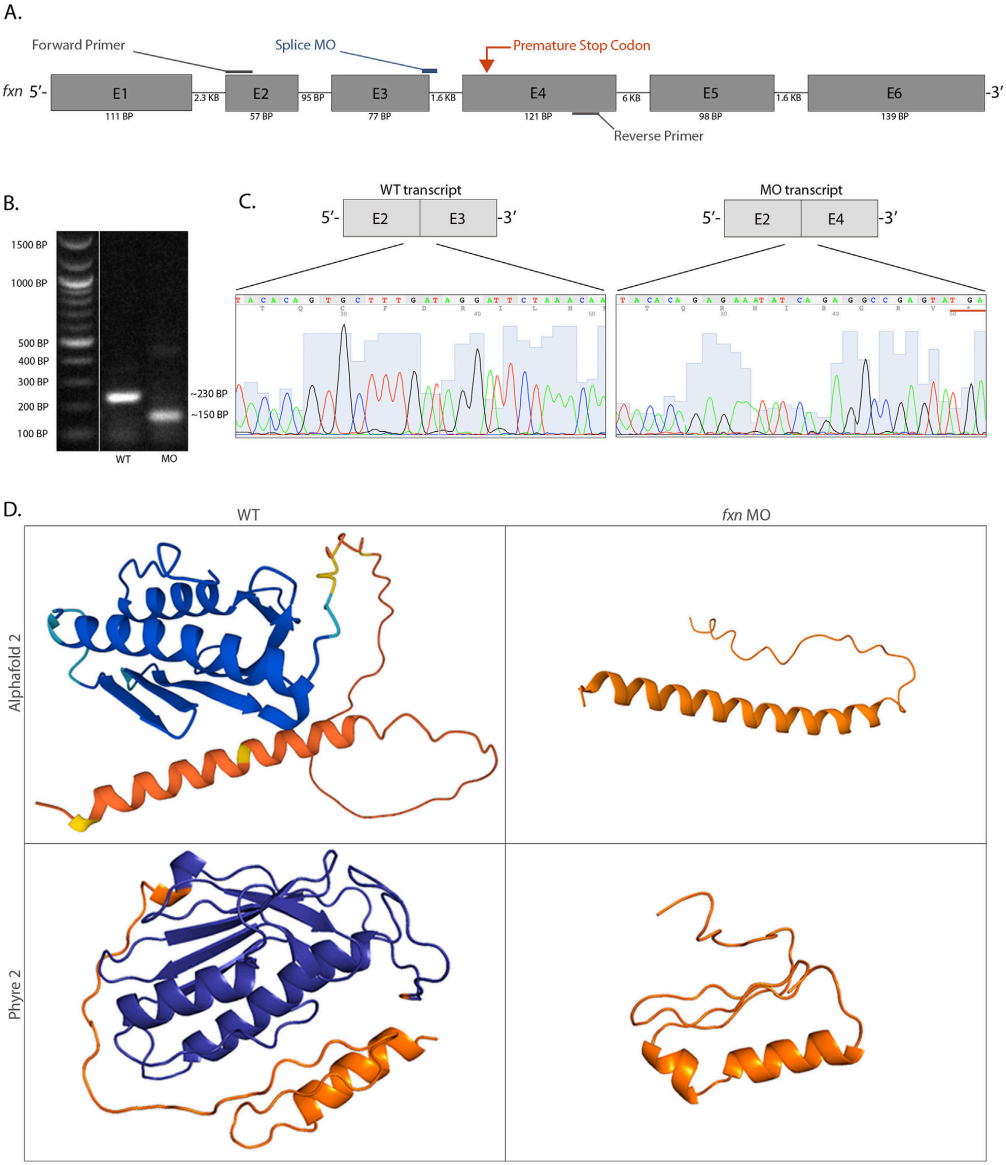
An antisense morpholino oligomer targeting the exon 3 and exon 4 splice region is sufficient to completely knockdown *fxn* expression in zebrafish. **(A)** A schematic of the *fxn* gene with annotations showing the morpholino and primer locations. **(B)** After cDNA synthesis using RT-PCR, analysis of the products through gel electrophoresis revealed that morphant animals produced a shorter *fxn* transcript and a wild-type product was absent. **(C)** Sanger sequencing data revealed that the *fxn* morphant transcript had a complete deletion of exon 3, which encoded an in-frame premature stop codon (underlined). **(D)** Three-dimensional protein folding predictions suggested a highly disrupted Fxn protein structure based on the altered mRNA sequence caused by splice interference due to the *fxn* morpholino. The endogenous Fxn consists of three alpha helices and five beta sheets, and the altered Fxn consists of an alpha helix and an interdomain loop. The orange refers to the mitochondrial trafficking sequence, an area of the protein that has low confidence for the folding prediction because of the lack of conservation between species in this specific domain. These folding predictions strongly suggest that significant regions of the Fxn protein are abrogated in the morphants.

To address this question, we developed a *fxn* knockdown zebrafish model using an antisense oligomer morpholino strategy to specifically target zygotic *fxn* expression, thereby preserving expression from maternal *fxn* transcripts. This model allows us to model Fxn deficiency in the zebrafish, rather than a complete Fxn absence, similar to what is seen in human patients that experience a reduction of FXN (Filla et al., 1996; Gellera et al., 2007). Our morpholino (MO) targets the exon 3 splice donor site, thus was predicted to interfere with proper connection between the exon 3 and exon 4 coding sequence (Figure 1A). Wild-type zebrafish were microinjected with *fxn* MO at the 1 cell stage, and at the 28 somite stage (ss) we employed reverse transcriptase polymerase chain reaction (RT-PCR) followed by gel electrophoresis (Figure 1B) and Sanger sequencing (Figure 1C) to scrutinize the effect on *fxn* transcripts. While wild-type controls exhibited normal splicing, embryos injected with the *fxn* exon 3 splice donor MO exhibited exon skipping. Morphant transcripts had a complete deletion of exon 3 and exon 2 was spliced onto exon 4 (Figure 1C). This aberrant splicing event resulted in a predicted frameshift of the open reading frame and the presence of an in-frame premature stop codon near the beginning of exon 4 (Figure 1C).

Next, using the *fxn* transcript sequences expressed by wild-type and *fxn* morphants, we employed the protein folding prediction algorithms Alphafold 2 and Phyre 2 to predict the three-dimensional structure of the Fxn protein (Figure 1D; Kelley et al., 2015; Jumper et al., 2021). This approach was utilized because at this time there is currently no x-ray crystallography data for zebrafish Fxn, and we wanted to visualize how the *fxn* transcript alteration would disrupt the Fxn protein structure. The two algorithms predicted similar structures for both the endogenous Fxn protein and the product of our zygotic Fxn knockdown, but with critical differences (Figure 1D). The endogenous immature Fxn protein predictions consist of three alpha helices and five beta-sheets (Figure 1D). This is consistent with the x-ray crystallography data of mature human Fxn which has two alpha helices and five beta-sheets (Dhe-Paganon et al., 2000). In contrast, the zygotic Fxn protein knockdown was predicted by the algorithms to have only one alpha helix (Figure 1D). Therefore, the algorithm predictions suggest that the morpholino would not only disrupt the three-dimensional structure of zebrafish Fxn, but indeed abrogate the active site due to the location of the premature stop codon.

### 2.2 Distinct FRDA phenotypes are present in *Fxn-*deficient zebrafish

In human patients with FRDA, multiple cell types are affected. Over time, there is considerable neuronal degeneration in the central and peripheral nervous systems and many patients experience severe hypertrophic cardiomyopathy, which is the most common cause of death (Koeppen and Mazurkiewicz, 2013; Koeppen et al., 2015). These neural and cardiac phenotypes have been recapitulated in *Fxn-*deficient mouse lines as well (Chandran et al., 2017).

**FIGURE 2:**
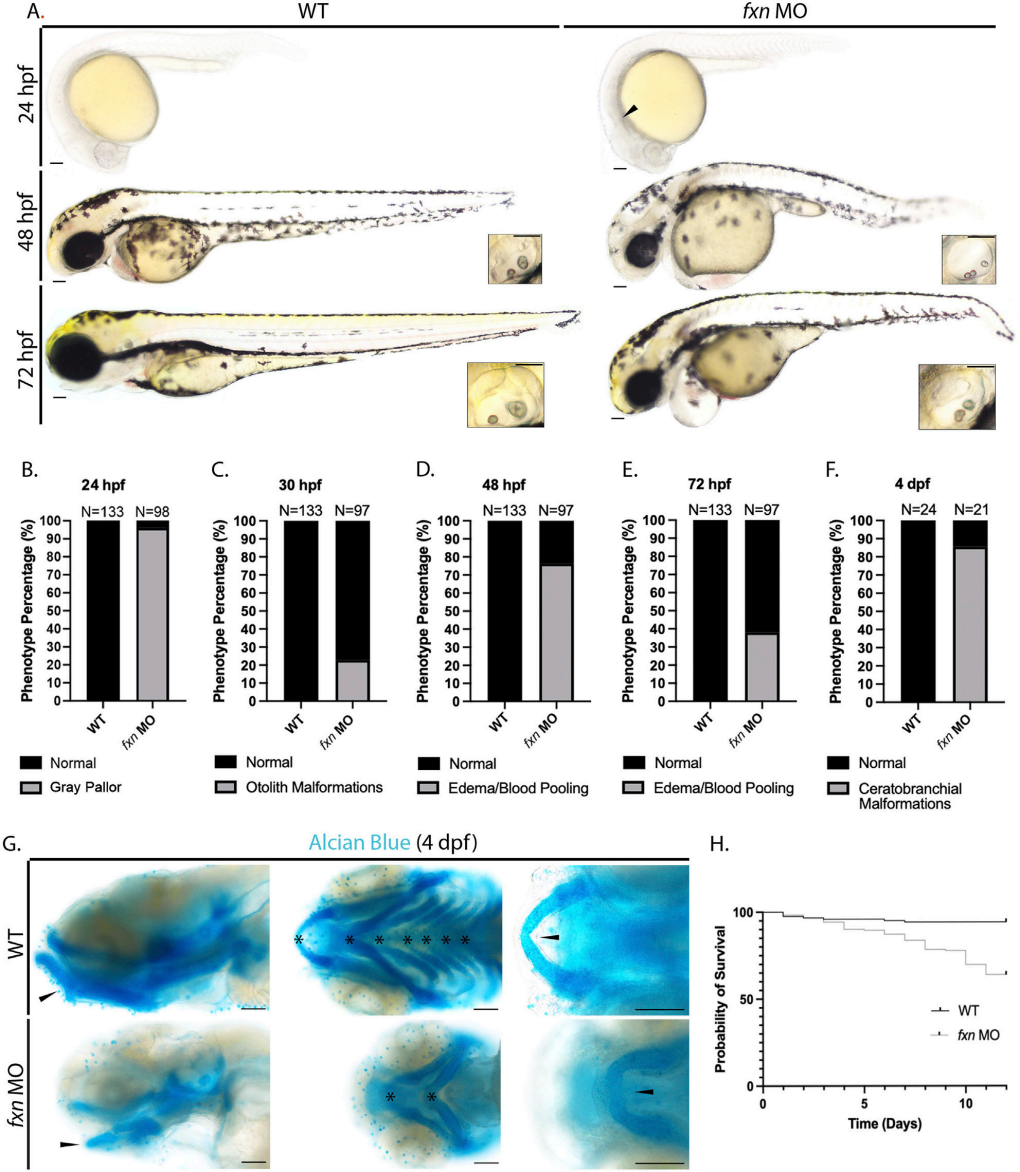
Morphological analysis of *fxn-*deficient zebrafish embryos reveal phenotypes indicative of several developmental malformations. **(A)** Brightfield microscopy revealed a diffuse gray pallor in the cranium of 24 hpf *fxn* morphants as well as blood pooling and pericardial edema at the 48 and 72 hpf stages. Insets show otolith malformations in the *fxn* morphants. Scale bars = 50 um. **(B–F)** Penetrance of observed phenotypes. **(B)** Gray pallor at 24 hpf. **(C)** Otolith malformations at 30 hpf. Edema/blood pooling at **(D)** 48 hpf and **(E)** 72 hpf. **(F)** Ceratobranchial malformations at 4 dpf. **(G)** Alcian blue staining revealed ceratobranchials 1-5 were missing in *fxn* morphants. Additionally, Meckel’s cartilage failed to fuse and the mandibular prominence did not curve ventrally as it does in wild-type animals. Scale bars = 50 um. **(H)** Kaplan-Meier survival curve documenting survival in wild-type embryos compared to *fxn* morphants. *Fxn* morphant animals displayed a 64% chance of survival compared to the 94% chance of their wild-type siblings during the first 12 days of life.

To explore and characterize development in *fxn-*deficient zebrafish, we first examined their live morphology at 24, 48, and 72 h post fertilization (hpf) (Figure 2A). We observed several striking phenotypes in *fxn* morphants compared to wild-type embryos. At 24 hpf, 96.3% of *fxn-*deficient embryos had a gray head pallor, a hallmark of cell death (Figures 2A,B). Also, 22.9% of the *fxn-*deficient embryos displayed hallmarks of malformed ear development, with three otoliths at 30 hpf compared to wild-type controls who formed otic vesicles with two otoliths (Whitfield, 2019) (Figures 2A,C). At 48 hpf, 76.4% of the *fxn-*deficient embryos had blood pooling in or near the pericardium which was often present with pericardial edema, the latter of which is one hallmark of renal dysfunction (Figures 2A,D). At 72 hpf, pericardial edema/blood pooling was present in 38.1% of the morphant animals (Figures 2A,D). Further, the majority of *fxn-*deficient embryos exhibited overt jaw malformations, where jaws were significantly smaller in size (Figure 2A). To better examine facial structure and jaw development, we performed Alcian blue staining to reveal the underlying cartilaginous cranial structures at 4 days post fertilization (dpf) (Figures 2F,G). We found that 85.7% of the morphants failed to develop ceratobranchials 1-5 by 4 dpf (Figures 2F,G). The mandibular prominence in these animals also had a convex curve when visualized laterally, rather than a concave curve that follows the profile of the maxillary prominence. Additionally, the Meckel’s cartilage also failed to fuse medially (Figure 2G). These data strongly suggest that *fxn* expression is required for the proper development of the pharyngeal arches and ceratobranchials.

Finally, we noted that as *fxn-*deficient zebrafish developed between 24 and 72 hpf, they displayed a shorter tip to tail length compared to wild-type embryos, suggestive that their growth was being compromised wherein they displayed a general failure to thrive (Figure 2A). Given this observation, we examined whether *fxn* deficiency would impact survival. Over the course of 12 days, *fxn-*deficient embryos exhibited a 64% chance of survival as opposed to wild-type controls that by comparison had a 94% chance (Figure 2H). These data strongly suggest that *fxn* deficiency disrupts the proper development of zebrafish embryos and their transition to a healthy larval stage of ontogeny.

### 2.3 *Fxn* is required for proper pronephros morphogenesis

The nervous system, heart, and kidneys have high metabolic rates. This would cause one to hypothesize that all of these tissues would be affected in FRDA patients as well as FXN-deficient mammalian models. Further, FXN is abundantly expressed in the embryonic and adult mouse kidney (Yue et al., 2014). However, kidney function has not been reported to be affected in the lifespan of FRDA patients (Reetz et al., 2018; Shinnick et al., 2016; Watters et al., 1981) and has similarly not been observed in animal models. This paradox, even more puzzling given the compromised fluid balance with severe edema observed in our *fxn-*deficient zebrafish embryos which are hallmarks of compromised renal physiology (Drummond et al., 1998; Wingert and Davidson, 2008; Gerlach and Wingert, 2013; Desgrange and Cereghini, 2015; Poureetezadi and Wingert, 2016; Fatma et al., 2021), led us to investigate next the formation and function of the zebrafish pronephros.

**FIGURE 3:**
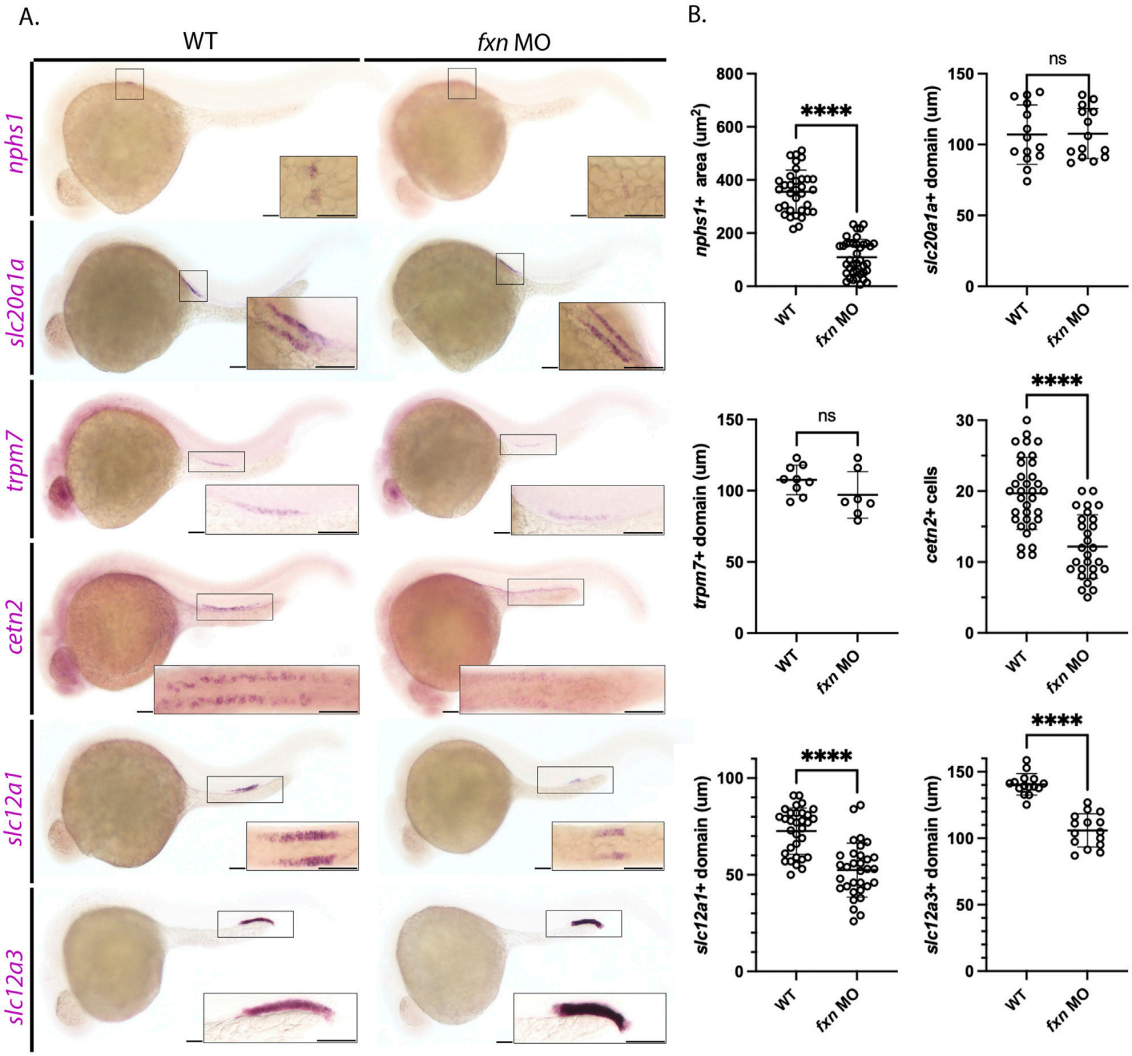
Pronephros segment development is significantly disrupted in *fxn-*deficient zebrafish embryos. **(A)** WISH experiments reveal that *fxn* is required for the proper formation of the podocytes, multiciliated cells (MCCs), distal early, and distal late tubule. The previously mentioned domains were visualized via the *nphs1,cetn4, slc12a1,* and *slc12a3* probes, respectively. Formation of the proximal convoluted and straight tubules, as assessed by expression of *slc20a1a* and *trpm7* revealed no significant differences between wild-type embryos and *fxn* morphants. Scale bars = 50 um. Each boxed lateral region in the embryos corresponds to the inset, which shows a dorsal view of that corresponding area for *nphs1*, *slc20a1a*, *cetn2*, and *slc12a1* or a lateral view of the corresponding area for *trpm7* and *slc12a3*. **(B)** Unpaired t-tests of the phenotypes revealed by WISH. ****p < 0.0001.

To do this, we utilized whole mount *in situ* hybridization (WISH) to visualize the different domains of the zebrafish pronephros at the 28 ss with previously established specific markers of each individual cell type (Figure 3). First, we used the *nphs1* probe to mark the podocytes, which are essential to form the blood filtration unit used by the embryonic nephrons (Kramer-Zucker et al., 2005). The area occupied by *nphs1* positive cells was decreased in *fxn-*deficient embryos compared to wild-type embryos (Figures 3A,B). Next, cells in the proximal convoluted tubule were marked with the probe for *slc20a1a* (Wingert et al., 2007). There was no statistically significant difference in the *slc20a1a* domain between the two groups (Figures 3A,B). Additionally, there was not a statistically significant difference in the proximal straight tubule domain, as marked with *trpm7* (Figures 3A,B) (Wingert et al., 2007). Interestingly, while there was not a statistically significant difference in the domain of *trpm7* positive cells, the *fxn-*deficient embryos appeared to be expressing less of this transcript, as the stain was fainter. Next, multiciliated cells (MCC) were marked with *cetn2* (Ma and Jiang, 2007). The number of *cetn2* positive cells was significantly decreased in *fxn-*deficient embryos compared to wild-types, suggesting that were fewer MCCs developed in the pronephros. Lastly, we examined the distal tubule, which is comprised of distal early cells that express *slc12a1* and distal late cells that express *slc12a3* (Wingert et al., 2007). Both distal segments were significantly shorter in length in the *fxn-*deficient embryos compared to wild-type controls (Figures 3A,B).

**FIGURE 4:**
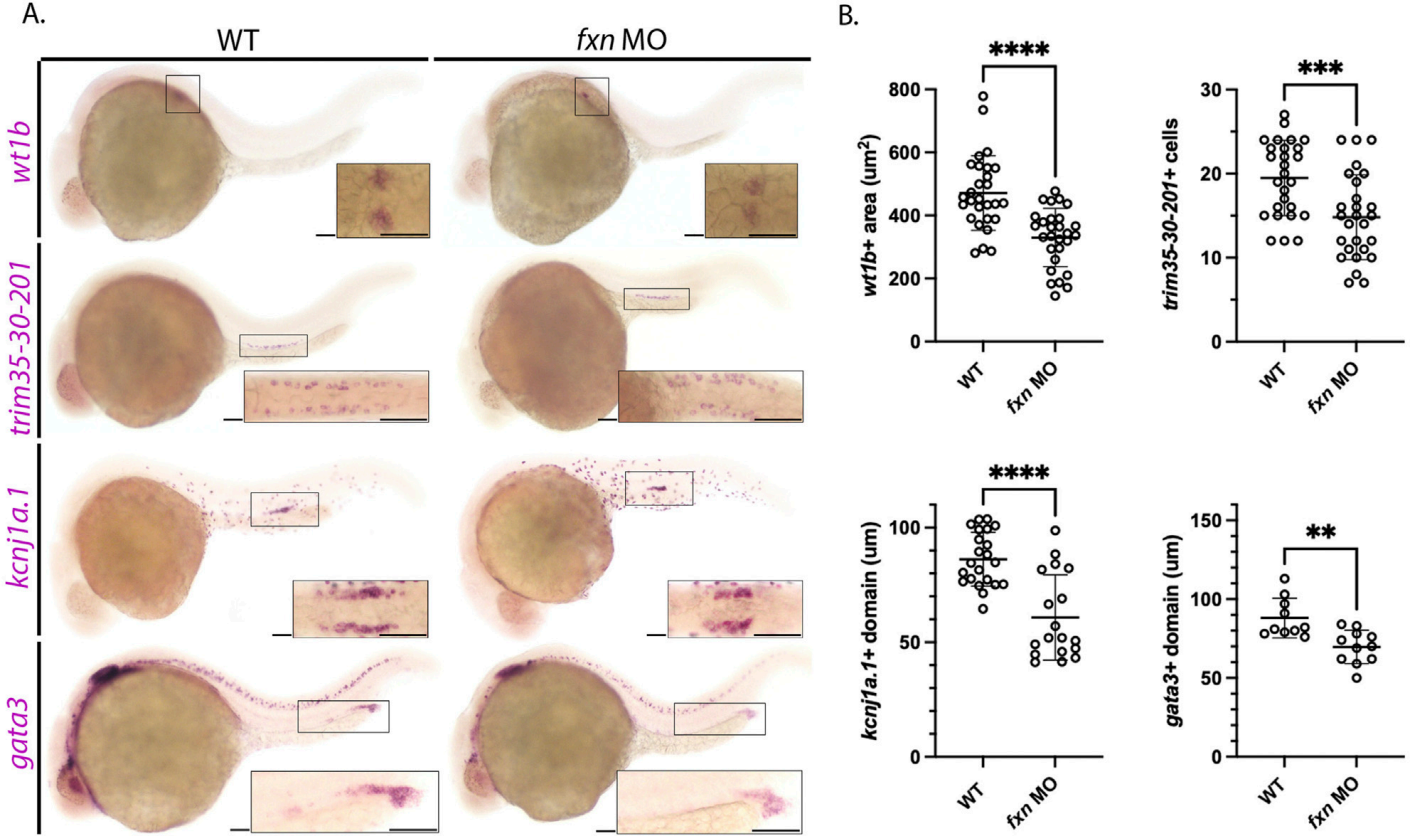
Further analysis of pronephros segment development in *fxn-*deficient zebrafish embryos confirms reductions in several cell populations. **(A)** WISH using the probes *wt1b, trim35-30-201, kcnj1a.1,* and *gata3* which mark the podocytes, multiciliated cells (MCCs), distal early and distal late domains respectively. *Fxn* morphants displayed a significant reduction in podocytes, reduction in MCC number, and reduced distal domains results with this set of markers, independently recapitulating the results shown in Figure 3. Scale bars = 50 um. Each boxed lateral region in the embryos corresponds to the inset, which shows a dorsal view of that corresponding area, with the exception of the *gata3* panels which show lateral views in the corresponding inset. **(B)** Unpaired t-tests demonstrating significantly affected pronephric domains in the *fxn* morphants. **p < 0.005, ***p < 0.0005, ****p < 0.0001.

To further assess each of the renal populations that were significantly altered in the *fxn-*deficient embryos, WISH was performed using a panel of independent molecular markers for the affected cell type (Figure 4). Specifically, we used *wt1b, trim35-30-201, kcnj1a.1,* and *gata3* to examine the podocytes, MCCs, distal early, and distal late, respectively (Figure 4). In each case, *fxn-*deficient embryos displayed significant reductions in the domain occupied by these pronephros populations compared to wild-type embryos, confirming our previous conclusions about the changes to each cell type based on our initial characterization of unique markers for each lineage (Figure 3).

Next, we utilized WISH to examine the emergence of the podocyte, distal early and distal late lineages at 22 ss and 26 ss, timepoints which precede the completion of nephron segmentation (Wingert et al., 2007; Wingert and Davidson, 2011). Additionally, these lineages were examined at 32 hpf to examine if the appearance of each population was developmentally delayed. The podocyte lineage was significantly reduced in *fxn-*deficient embryos compared to wild-types at the 22 ss, 26 ss and 32 hpf (Supplemental Figure S1). Likewise, the distal early lineage as marked by *slc12a1* and distal late lineage as marked by *slc12a3* was significantly reduced in *fxn-*deficient embryos compared to wild-type controls (Supplemental Figures S2, S3). Taken together, these data suggest that *fxn* is critical for the proper emergence of multiple lineages within the zebrafish pronephros.

To further explore this notion, we performed several control studies. First, we examined whether the *fxn* morpholino might have off target effects, which can be associated with edema and grey cranial pallor. The p53 morpholino is well known to attenuate common off target effects of morpholinos (Robu et al., 2007). We observed that co-injection of the p53 morpholino with the *fxn* morpholino led to similar phenotypes as *fxn* morpholino alone (Supplemental Figure S4), namely gray pallor in the cranium at 24 h post fertilization and edema between 48 and 72 hpf. In light of these results, we conclude that off target effects are not a significant component of the *fxn-*deficient morphant phenotypes. Additionally, wild-type zebrafish were microinjected with a standard control morpholino, raised to 24 hpf, and fixed for nephron segment analysis. WISH revealed that formation of the podocytes and tubule segments were not altered due to the standard morpholino control (Supplemental Figure S5). Taken together, these studies lead us to conclude that fxn deficiency causes specific defects in development including pronephros formation.

### 2.4 MCCs are significantly decreased at several different time points

**FIGURE 5:**
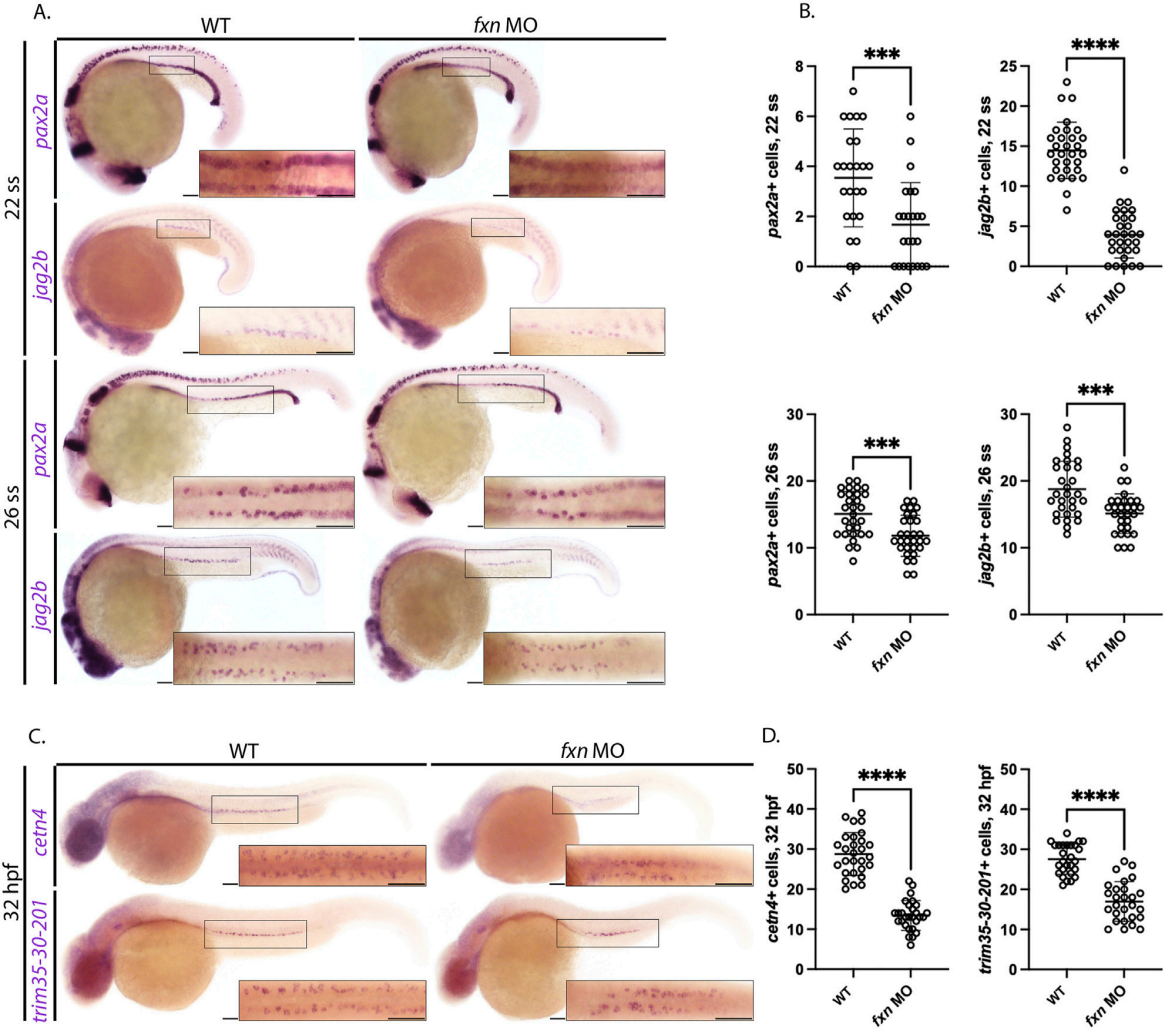
*Fxn-*deficient zebrafish embryos form reduced multiciliated cell (MCC) progenitors. **(A)** WISH analysis at the 22 and 26 ss utilizing the probes *pax2a* and *jag2b* revealed that the population of MCC progenitors was significantly reduced in *fxn* morphants compared to wild-type control embryos. Scale bars = 50 um. **(B)** Unpaired t-test of the corresponding WISH experiments in panel **(A)**. **(C)** WISH analysis at the 32 hpf time point utilized the probes *trim35-30-201* and *cetn4,* which are expressed by differentiating MCCs, and revealed that MCC numbers were still significantly reduced at this later timepoint. Scale bars = 50 um. Each boxed lateral region in the embryos corresponds to the inset, which shows a dorsal view of that corresponding area. **(D)** Unpaired t-test of the corresponding WISH experiments in panel **(C)**. ***p < 0.0005, ****p < 0.0001.

Defects in MCC development have been linked to embryonic fluid imbalance such as pericardial edema (Marra and Wingert, 2016; Marra et al., 2019; Chambers et al., 2020b). Therefore, we next sought to assess MCC specification in *fxn-*deficient embryos and examined whether the MCC populace was diminished at later timepoints. To characterize MCC progenitors, we examined the expression of *pax2a* and *jag2b* at the 22 ss using WISH and quantified these populations (Marra et al., 2019). MCC progenitor numbers in *fxn-*deficient animals were significantly decreased as compared to wild-type controls (Figures 5A,B). To examine whether the MCC populations might be diminished due to developmental delay, WISH was also performed several hours subsequent to this time point to survey the number of *pax2a* and *jag2b* expressing MCC progenitors. *Fxn-*deficient embryos at the 26 ss had significantly decreased *pax2a+* and *jag2b +* MCCs compared to wild-type controls (Figures 5A,B). These data further support the conclusion that MCCs are not specified correctly in the absence of Fxn function.

To examine the progression of MCC development in the context of *fxn* deficiency, we conducted WISH analysis on 32 hpf animals using the *trim35-30-201* and *cetn2* riboprobes, which mark differentiating MCCs (Ma and Jiang, 2007; Liu et al., 2007; Marra and Wingert, 2016). While both the wild-types and *fxn-*deficient embryos formed MCCs that expressed these more mature markers, consistent with the ability of this lineage to differentiate, the number of *trim35-30-201+* and *cent2+* MCCs were significantly reduced in number in *fxn-*deficient embryos compared to wild-types (Figures 5C,D). In sum, these findings suggest that fewer MCCs are patterned in the *fxn-*deficient pronephros.

### 2.5 The pronephros in *Fxn-*deficient zebrafish is dysfunctional

**FIGURE 6:**
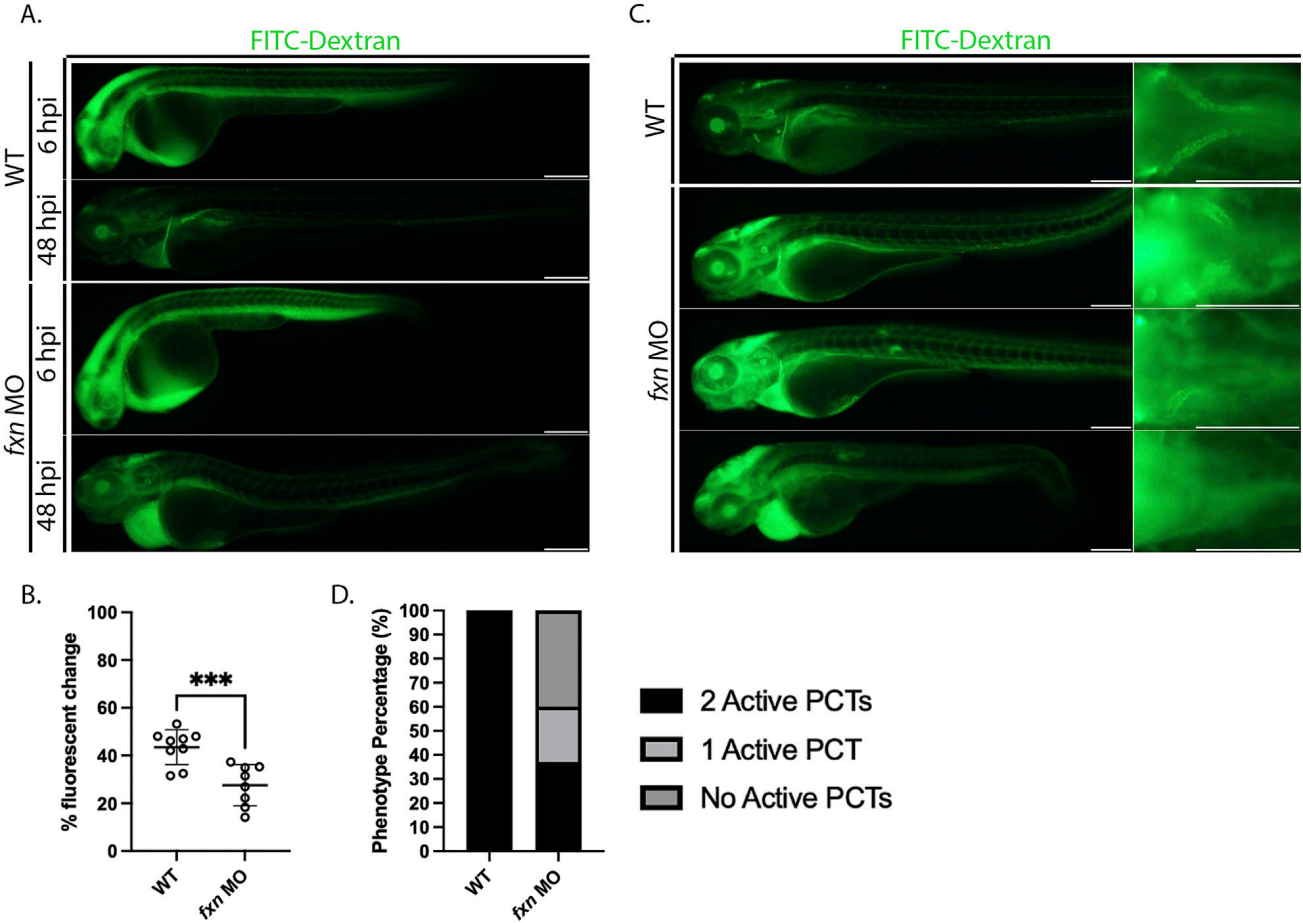
*Fxn-*deficient zebrafish embryos display renal clearance defects and some proximal reabsorption defects. **(A)** *fxn* morphants failed to excrete the FITC-Dextran at the same rate as their wild-type siblings. Images of the same wild-type and *fxn-*deficient animals 6 hours and 48 h after injection with the FITC-dextran. FITC-Dextran (40 kDa) was injected around 36 h post-fertilization. **(B)** An unpaired t-test of the percent fluorescent change of each animal. Percent fluorescent change was calculated using the 6 h post injection (hpi) and 48 hpi fluorescent intensity of the same animal. **(C)** Morphants exhibit decreased endocytosis activity in the proximal convoluted tubule (PCT). Among the morphants, there were three primary phenotypes. Morphant animals either had endocytosis activity in two PCTs, one PCT, or neither. These findings suggest that dysfunction within the PCT segments is one contributor to the fluid imbalance observed in *fxn-*deficient animals. **(D)** The proportions of proximal convoluted tubule phenotypes in the wild-type and *fxn* morphant groups. Scale bars are 200 um. ***p < 0.0005.

Because we observed a variety of renal cell populations being affected by *fxn-*deficiency, we hypothesized that pronephros dysfunction may indeed underlie the edema exhibited by *fxn-*deficient embryos. To test this hypothesis, we injected 36 hpf wild-type controls and *fxn-*deficient embryos and with 40 kD FITC-Dextran, as this compound can be utilized as a proxy to visualize and measure fluid clearance within live animals in real time (Weaver et al., 2022) (Figure 6). After introduction of this tracer, embryos were imaged at 6 h post injection (hpi) and 48 hpi to assess fluorescent intensity (Figure 6A). As observed previously, *fxn-*deficient embryos developed pronounced pericardial edema over this time period (Figure 6A). Upon analysis of the percentage fluorescent change between 6 and 48 hpi, *fxn-*deficient embryos displayed edema a significantly decreased ability to clear the FITC-dextran compared to wild-types (Figure 6B).

We also used FITC-Dextran to visualize proximal convoluted tubule (PCT) endocytosis activity. (Figure 6C). Receptor mediated endocytosis is utilized extensively by the proximal tubule to reabsorb materials in the renal filtrate (Anzenberger et al., 2006). While wild-type embryos exhibited robust endocytosis in the PCT of both nephrons, the *fxn* morphants displayed a range of reduced PCT function. Approximately 35% of *fxn* morphants had PCT endocytosis activity in both nephrons. However, about 25% of *fxn* morphants had PCT endocytosis activity in nephron only and about 40% of the *fxn* morphants had no PCT endocytosis in either nephron (Figure 6D). From this, we conclude that nephron function is compromised, and thus concluded that Fxn function is necessary to achieve proper pronephros function during development.

### 2.6 Cell death is amplified in *Fxn-*deficient zebrafish

Decreased expression of FXN leading to ROS and consequent cell death is hypothesized to be a major underlying pathogenic component of FRDA, commonly affecting the nervous system (Abeti et al., 2016; Koeppen et al., 2011; Koeppen and Mazurkiewicz, 2013). In our morphological studies, we had observed gray pallor in the developing central nervous system of *Fxn-*deficient embryos, which is a harbinger of cell death (Cheng et al., 2015). Thus, next we sought to characterize cell survival in *fxn-*deficient zebrafish to test the hypothesis that *Fxn* loss of function was indeed causing an increase in cell death across tissues during embryogenesis.

**FIGURE 7:**
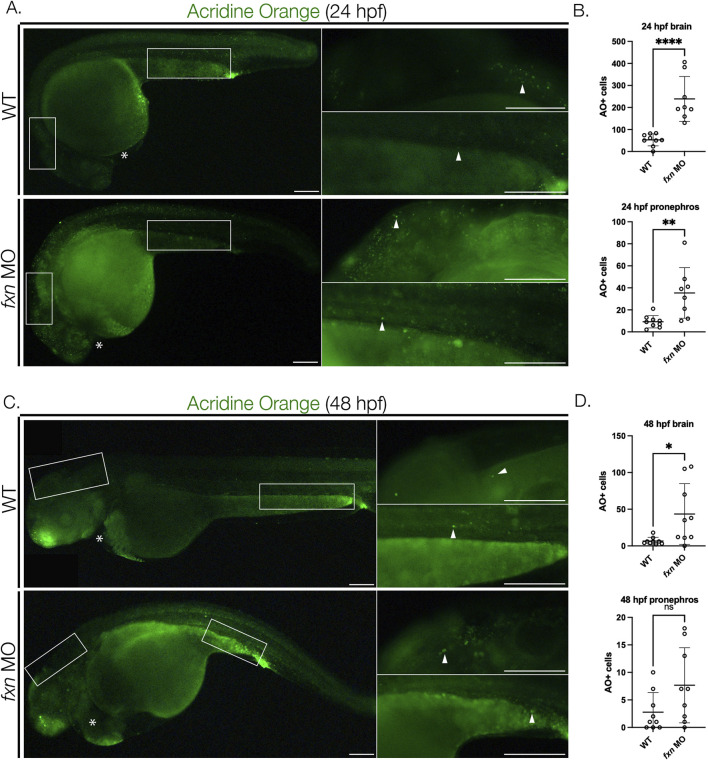
Cell death is elevated in *fxn-*deficient zebrafish embryos. **(A)** Images of AO-stained animals at 24 hpf. *Fxn* morphants animals have significantly increased cell death in the brain and pronephros at 24 hpf and increased cell death in the brain at 48 hpf. White boxes denote the locations for the panels on the right top (brain) and right bottom (pronephros). Scale bars = 50 um. **(B)** Unpaired t-tests of cell death in the brain and pronephros at 24 hpf. **(C)** Images of AO-stained animals at 48 hpf. White boxes denote the locations for the panels on the right top (brain) and right bottom (pronephros). Within the right top brain panel, each white box indicates a single positive cell. In *fxn* morphants, areas of the brain commonly had multiple cells as indicated by the white box. Scale bars = 50 um. **(D)** Unpaired t-tests of cell death in the brain and pronephros at 48 hpf. *p < 0.05, **p < 0.005, ****p < 0.0001.

To test this, we used 24 and 48 hpf *Fxn-*deficient embryos to perform acridine orange (AO) staining (Tucker and Lardelli, 2007), which is a DNA intercalating fluorescent dye that rapidly and robustly labels apoptotic cells and readily penetrates deep tissues in whole mount embryos (van Ham et al., 2010). Next, we quantified cell death in several regions, focusing on the brain as well as the pronephros and the heart. *Fxn-*deficient zebrafish had significantly more AO + cells in the brain at both 24 and 48 hpf compared to wild-types (Figures 7A–D). *Fxn-*deficient embryos also had significantly more AO + cells in the pronephros at 24 hpf than wild-type controls (Figures 7A,B). At the 48 hpf time point, however, there was not a significant difference in AO + cell number between wild-types and *Fxn-*deficient embryos (Figures 7C,D). Interestingly, we did not observe AO + cells within the heart in either wild-type or *fxn-*deficient embryos at 24 or 48 hpf (Figure 7). These observations suggest that cell death is a component of the developmental defects which transpire during both pronephros and central nervous system formation, but not the heart, in *fxn-*deficient zebrafish.

### 2.7 Pronephros segment formation is differentially compromised in fxn-deficient embryos

**FIGURE 8:**
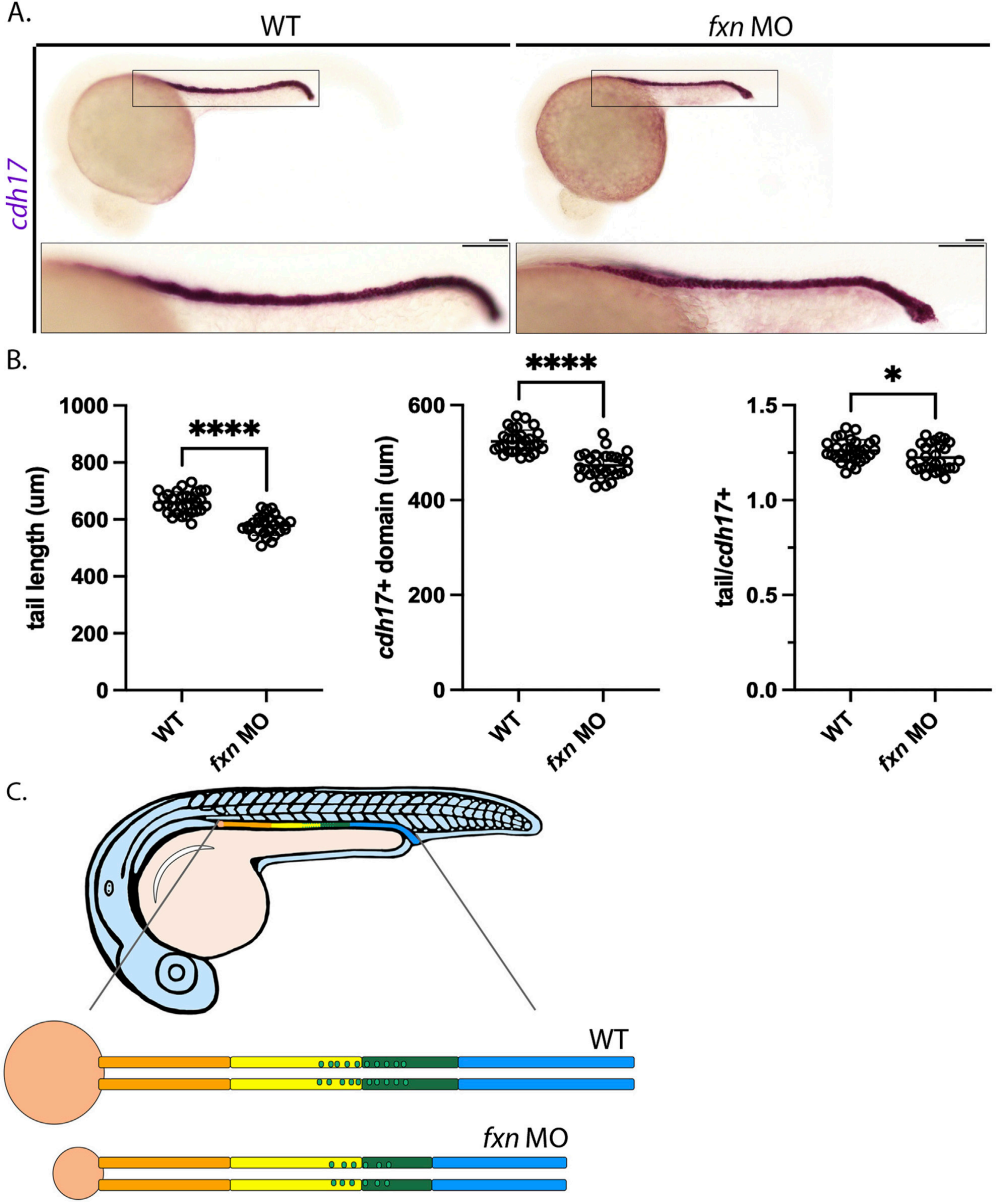
Tail/pronephros tubule ratio comparisons indicate that the embryonic kidney that develops within *fxn-*deficient zebrafish embryos was decreased in length proportionally to the decreased in tail length. **(A)** WISH analysis of pronephros tubule formation as marked by *cdh17* revealed that this structure was shorter in *fxn* morphants than it is in their wild-type siblings (scale bars = 50 um). Each boxed lateral region in the embryos corresponds to the inset, which shows an enlarged lateral view of that corresponding area. **(B)** Unpaired t-tests of tail length and pronephros length as marked by *cdh17* revealed that the length of both structures was significantly shorter in the morphants than in their wild-type siblings. An unpaired t-test of the ratios of tail and pronephros length also showed a statistically significant difference. *p < 0.05, *p* ****p < 0.0001. **(C)** *fxn* deficiency leads to formation of a pronephros which is decreased in size in porportion to the reduction in embryo size, however the distal segments are disporportionally reduced suggesting these lineages are uniquely affected by the loss of Fxn activity.

As noted previously, we found that *fxn-*deficient embryos were typically smaller in tip to tail length than wild-types of a comparable developmental age, suggesting compromised growth over time (Figure 2). Thus, we wondered whether the decreases seen in the several pronephros populations like the distal tubules and MCCs were simply due to the animal being smaller. To examine this possibility, we conducted WISH using *cdh17*, which is a pronephros specific marker (Figure 8). *cdh17* stains the length of the nephron tubules (Wingert et al., 2007), thus allowing us to compare the absolute length of the pronephros versus the embryo tail length from tip to tail (Figure 8A). By measuring and comparing these lengths in 24 hpf wild-types and *fxn-*deficient embryos, we found that the pronephros and the tail were both significantly shorter than in *fxn-*deficient embryos compared to wild-type control embryos at this age (Figure 8B). When we calculated the ratio of tail/*cdh17* length, *fxn-*deficient embryos were found to have a reduction in pronephros size that was slightly less severe than the decrease in tail length (Figure 8B). This suggests that the pronephros undergoes a decrease in size that is very similar to the overall embryonic size decrease in *fxn-*deficient embryos. We also examined performed tail analysis to explore whether the decreases in distal tubule length were proportionate to the smaller pronephros size. Examination of two distal early markers (*slc12a1, kcnj1a.1*) and two distal late markers (*slc12a3, ptgs1*) revealed that the decreased lengths of these segments were approximately 25% while tail lengths were reduced by approximately 10%–12% (Supplemental Figure S6). This analysis reveals that the distal segments of *fxn-*deficient embryos are disproportionately reduced compared to the tail or the nephron overall (Figure 8C). Future studies are needed to ascertain whether changes in regions such as the cloaca, collecting duct or Corpuscles of Stannius, endocrine glands associated with the pronephros lineage, are affected in *fxn-*deficient embryos and how changes in these cell types relate to the distal segment reductions.

## 3 Discussion

The roles of FXN in mammalian embryogenesis remain poorly understood. Here, we have created a new loss of function model for Fxn zygotic deficiency in the zebrafish, and utilized this paradigm to begin exploring the effects of diminished Fxn expression during vertebrate organogenesis. These studies have revealed that Fxn is required for the proper development of otolith sensory structures, pharyngeal arches, ceratobranchials, and several renal lineages that comprise the nephron functional units within the pronephros. We found that cell death is significantly increased during Fxn deficiency in both the central nervous system and pronephros—an observation which correlates with the finding that pronephros formation is differentially compromised in Fxn-deficient embryos. Lastly, we found that Fxn-deficient zebrafish embryos had an impaired ability to clear fluids. In part, fluid imbalance in *Fxn*-deficient zebrafish correlates to alterations in PCT reabsorption, which was compromised in ∼60% of *Fxn*-deficient embryos. Collectively, these studies have documented a number of significant phenotypic consequences for the loss of Fxn expression during early embryonic stages of zebrafish development.

Interestingly, several of the tissues that are affected by Fxn deficiency during zebrafish development are also affected in animal models of FRDA and human patients as well. For example, FRDA mouse models and patients experience neuron attrition or increased cell death during neuronal differentiation (Koeppen, 2011; Santos et al., 2001; Simon et al., 2004), and in the present study we found that neuron survival was reduced in Fxn-deficient zebrafish embryos at 24 and 48 hpf. Although the cause of death is unknown, Fxn-deficient zebrafish embryos had a lower chance of survival than their wild-type siblings, which parallels shorter life expectancies in mouse models (Perdomini et al., 2014) and humans (Andermann et al., 1976).

Contrary to our findings in this report, problems stemming from craniofacial malformations, otoliths, pharyngeal arches, and ceratobranchial malformations have not been reported in other model organisms or FRDA patient studies. However, one study demonstrated that zebrafish otoliths, pharyngeal arches, and ceratobranchials are all derived from the neural crest (Kague et al., 2012). This may correlate with the study which demonstrated that apoptosis is increased in differentiating neuroectoderm (Santos et al., 2001).

In addition, alterations in kidney form and function have not been previously associated with FXN deficiency in other models or humans. Thus, our findings suggest for the first time that Fxn has roles in supporting embryonic kidney development. Future studies are necessary to explore whether Fxn has direct or indirect roles in the emergence, differentiation and survival of the intermediate mesoderm in higher vertebrates. Our work implicates Fxn as requisite for proper survival of renal populations. More research is needed to further understand the dynamics of cell loss, such as the mechanisms of cell death, and whether cell proliferation is compromised. The latter may be relevant given the early population changes in the podocyte, MCC and distal segments, and our observations that pronephros growth is differentially compromised in the context of Fxn deficiency.

In conclusion, we have created a zebrafish model of zygotic Fxn deficiency that can be utilized moving forward to expand our fundamental understanding about the developmental roles of *fxn*. This model is particularly well suited to examine the effects of Fxn during early ontogeny and organogenesis. Future studies are needed to create complementary genetic models of *fxn* loss of function using tools such as CRISPR/Cas9, and to investigate molecular changes associated with reduced Fxn levels. For example, Fxn dysfunction is closely associated with mitochondrial pathologies, which were not examined in the present work. Overall, our hope is that the fxn deficiency model described here will prove useful to other FRDA researchers in the collective effort to understand the effects of decreased FXN expression in humans, and to assist with efforts to identify new therapeutics to treat this devastating condition.

## 4 Materials and methods

### 4.1 Zebrafish husbandry

Zebrafish were cared for by the Freimann Life Sciences animal facility. All studies were approved by the University of Notre Dame Institutional Animal Care and Use Committee (IACUC), under protocol numbers 19–06-5,412 and 22–07-7,335. The Tübingen strain was used for all our zebrafish studies. Embryos were incubated at 28°C in E3 embryo media. Once the animals reached the relevant developmental stage (Westerfield, 1993), they were used for live imaging or anesthetized with 0.02% tricaine and then fixed with 4% paraformaldehyde/1x phosphate buffered saline for gene expression studies (Kimmel et al., 1995).

### 4.2 Morpholino knockdown

Morpholinos were obtained from Gene Tools, LLC. The morpholino sequence targeting the zebrafish *fxn* Exon 3/Exon4 splice site is 5′-CTA​AAT​TTA​CCT​GAG​GTG​ATG​TGC​C-3’. The morpholino was injected into zebrafish embryos at the 1 cell stage and uninjected wild-type siblings were raised as controls. The concentration and volume used are 3 g/L and ∼1.5 nL respectively. The standard control morpholino used was 5′-CCT​CTT​ACC​TCA​GTT​ACA​ATT​TAT​A-3’. The p53 morpholino used was 5’ –AGA​ATT​GAT​TTT​GCC​GAC​CTC​CTC​T-3’.

### 4.3 RT-PCR and sanger sequencing

RNA was extracted from approximately 30 animals from wild-type and morphant groups using TRIZOL Invitrogen (15596026). Next, cDNA was generated using qScript cDNA Supermix (Quantabio). The primers used were: forward 5′- TCA​ATA​AGT​GGT​GGT​AGG​AGT​GTT​T-3′, and reverse 5′-CCA​GTG​AAG​TTT​TCA​TCT​GTG​AGA​T-3’. Our samples were sequenced at the University of Notre Dame Genomics and Bioinformatics Core Facility. The trace files were analyzed using 4Peaks (4Peaks by A. Griekspoor and Tom Groothuis, nucleobytes. com.)

### 4.4 Whole-mount *in situ* hybridization (WISH)

WISH was performed in the same manner as described by previous publications (Chambers B. E. et al., 2020; Chambers J. M. et al., 2020; Marra et al., 2019). The antisense RNA, digoxigenin-labeled probes that were used are *cdh17, wt1b, nphs1, slc20a1a, trpm7, trim35-30-201, cetn4, pax2a, jag2b, slc12a1, kcnj1a.1, slc12a3,* and *gata3*. These probes were generated using *in vitro* transcription from IMAGE clone templates as described by previous publications (Gerlach and Wingert, 2014; Wingert et al., 2007).

### 4.5 FITC-dextran injections

Wild-type and *fxn* morphant animals were incubated in 0.003% phenylthiourea (Sigma-Aldrich, P7629) starting at 8 hpf. Animals were anesthetized at 36 hpf with 0.02% tricaine and subsequently injected with 3 nL of 40 kDa FITC-Dextran (Invitrogen, D-1845) (5 mg/mL). The injection site was around somite three in muscular tissue as described in a previous publication (Kroeger et al., 2017). The animals were then imaged at 6- and 48-hours post injection. The fluorescent change of the entire animal was measured using the FIJI software. The percent change of fluorescent intensity was calculated for each animal by keeping animals separate in a 96-well plate. The change in fluorescent intensity for both groups was analyzed using an unpaired t-test.

### 4.6 Acridine orange (AO)

Cell death was approximated using AO (Sigma-Aldrich A6014) as described in previous publications (Kroeger et al., 2017; Weaver et al., 2022). 50 mg of AO were dissolved into 50 mL of MilliQ water as a stock solution (100x). The stock solution was stored at −20 C in a light-protected container. AO was diluted down to a 1x concentration in E3 embryo medium before being applied to 24 and 48 hpf animals which had previously been incubated in 0.003% phenylthiourea starting at 8 hpf. Animals were incubated for 30 min at room temperature in the AO/E3 solution and then washed three times over the course of 10 min in E3. Samples were then anesthetized in a tricaine/E3 solution and immediately imaged in methylcellulose.

### 4.7 Alcian blue (AB)

AB (Thermofisher 05500-5G) staining was conducted as described in a previous publication (Neuhauss et al., 1996; Weaver et al., 2022). Wild-type and *fxn* morphants were fixed at 4 dpf using 4% paraformaldehyde at 4 C for 16 h. Dehydration was done using 100% methanol at −20 C. The rehydration was done with a series of methanol washes of decreasing concentration. Animals were bleached for 2 h, rinsed in PBST, and then digested using a proteinase K (Roche 3115836001) (10 mg/mL for 15 min). Animals were then rinsed in PBST again and incubated at room temperature on a rocker for 16 h in an Alcian Blue (0.1%), ethanol (70%), and HCl (5%) solution. The animals were then destained on a rocker in an ethanol (70%) and HCL (5%) solution for 8 h. After destaining, the animals were washed in PBST, dehydrated in a series of ethanol washes that increased in concentration, and stored in glycerol prior to imaging.

### 4.8 Image acquisition

WISH, AB, AO, and live animal images were all taken on a Nikon Eclipse microscope with a DS-Fi2 camera. Methylcellulose with small amounts of tricaine was used to hold the animals in place for the live images and glycerol was used to hold the fixed animals.

### 4.9 Quantification and statistical analysis

All measurements were taken using the Nikon Elements imaging software on 10x images. Surface area measurements were taken using the auto-select tool. Length measurements were taken using the polyline tool. MCCs were counted manually using the binoculars on the microscope. All of the WISH data were entered into GraphPad Prism and analyzed using unpaired t-tests. AB data was analyzed using the FIJI software. AO images were analyzed by counting fluorescent cells manually. Live, brightfield images were analyzed by quantifying the phenotypes manually.

## Data Availability

The original contributions presented in the study are included in the article/supplementary material, and further inquires can be directed to the corresponding authors.
