# Supplementary material for: Frataxin is essential for zebrafish embryogenesis and pronephros formation
Source: Front Cell Dev Biol. 2024 Dec 11;12:1496244. doi: 10.3389/fcell.2024.1496244 (PMC11669007; doi:10.3389/fcell.2024.1496244)
Supplement: Supplementary file 1 [file DataSheet1.docx]

***Supplementary Material***

***frataxin* is essential for zebrafish embryogenesis and pronephros formation**

**Wesley S. Ercanbrack^1*^, Austin Dungan^2^, Ella Gaul^2^, Mateo Ramirez^2^, Alexander J. DelVecchio^2^, Calvin Grass^2^, Rebecca A. Wingert^1*^**

*** Correspondence:**

Rebecca A. Wingert
rwingert@nd.edu

Wesley S. Ercanbrack

wercanbr@nd.edu

**
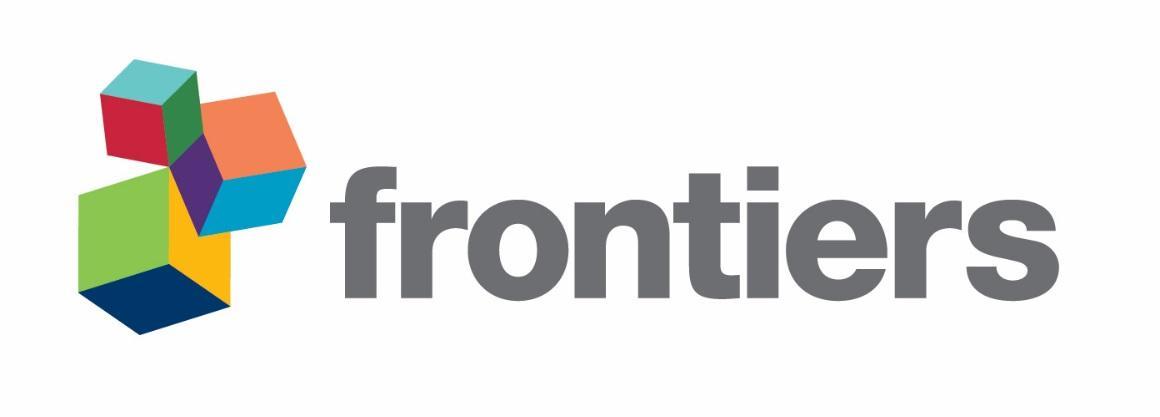
**


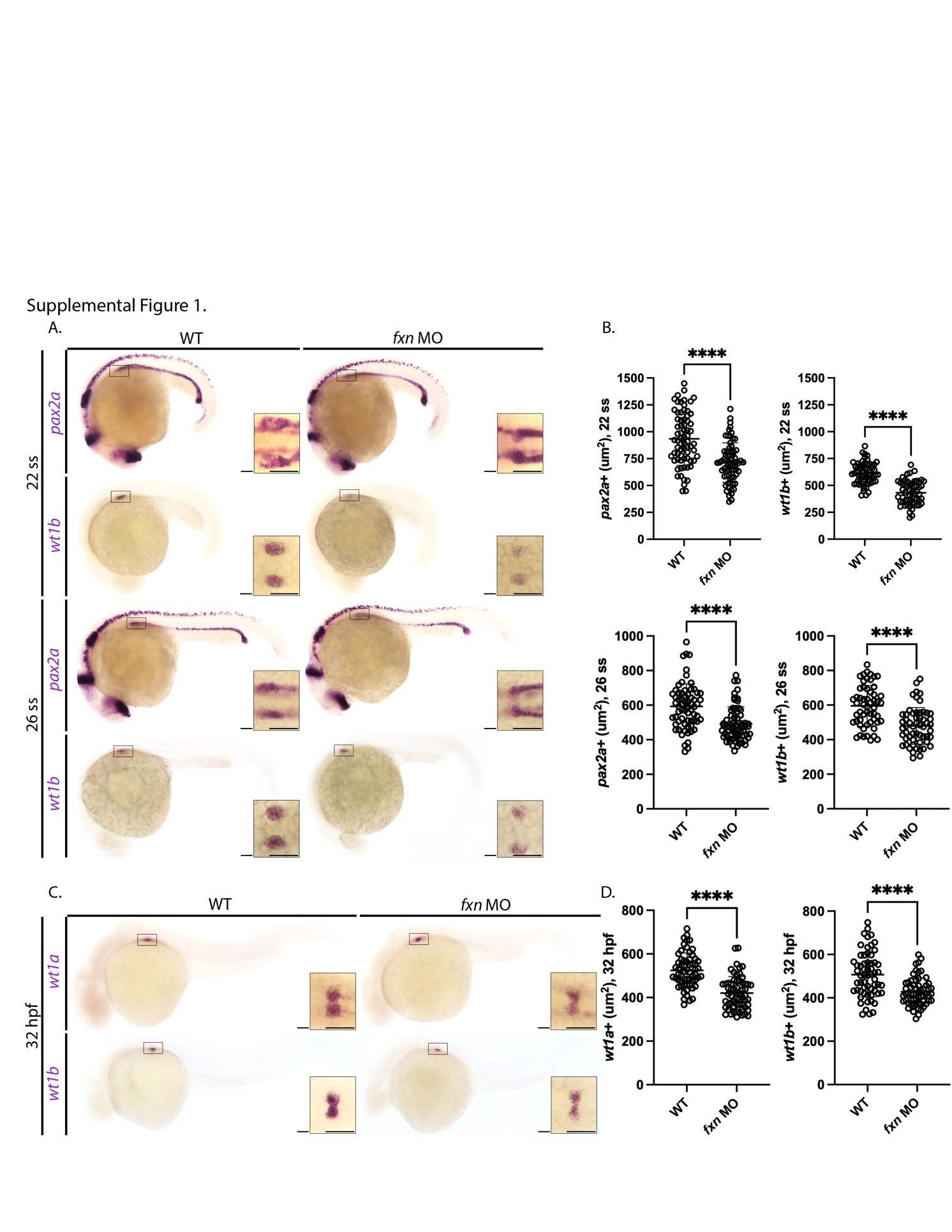
 **Supplementary Figure 1.** WISH reveals reduced podocyte surface area at 22 ss, 26 ss, and 32 hpf. A) The experiments at the 22 ss and 26 ss utilized probes *pax2a* and *wt1b*. Scale bars = 50 um. B) Unpaired t-test of the corresponding WISH experiments in panel A. C) The WISH experiments at the 32 hpf time point utilized the probes *wt1b* and *wt1a*. The probe *pax2a* was not used at this time point because it localizes to the neck of the pronephros around this stage. Scale bars = 50 um.  D) Unpaired t-test of the corresponding WISH experiments in panel C.


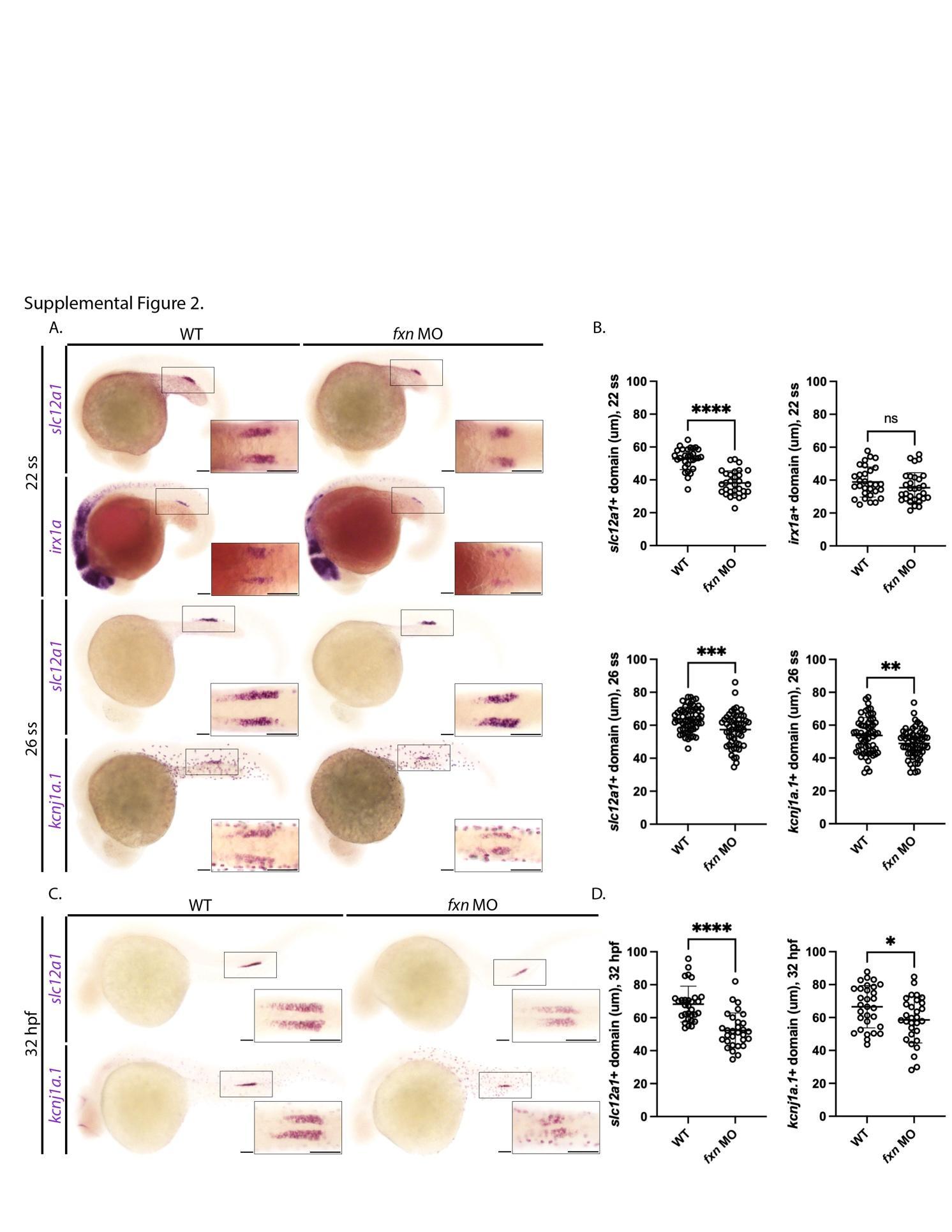
**Supplementary Figure 2.** WISH reveals reduced DE length at 22 ss, 26 ss, and 32 hpf. A) The experiments at the 22 ss utilized probes *slc12a1* and *irx1a.* There is no statistical difference between groups using the *irx1a* probe at the 22 ss. The experiments at the 26 ss utilized the probes *slc12a1* and *kcnj1a.1*. The probe *irx1a* was used at the 22 ss instead of *kcnj1a.1* because the latter is not expressed at 22 ss. Scale bars = 50 um. B) Unpaired t-test of the corresponding WISH experiments in panel A. C) The WISH experiments at the 32 hpf time point utilized the probes *slc12a1* and *kcnj1a.1*. Scale bars = 50 um.  D) Unpaired t-test of the corresponding WISH experiments in panel C.


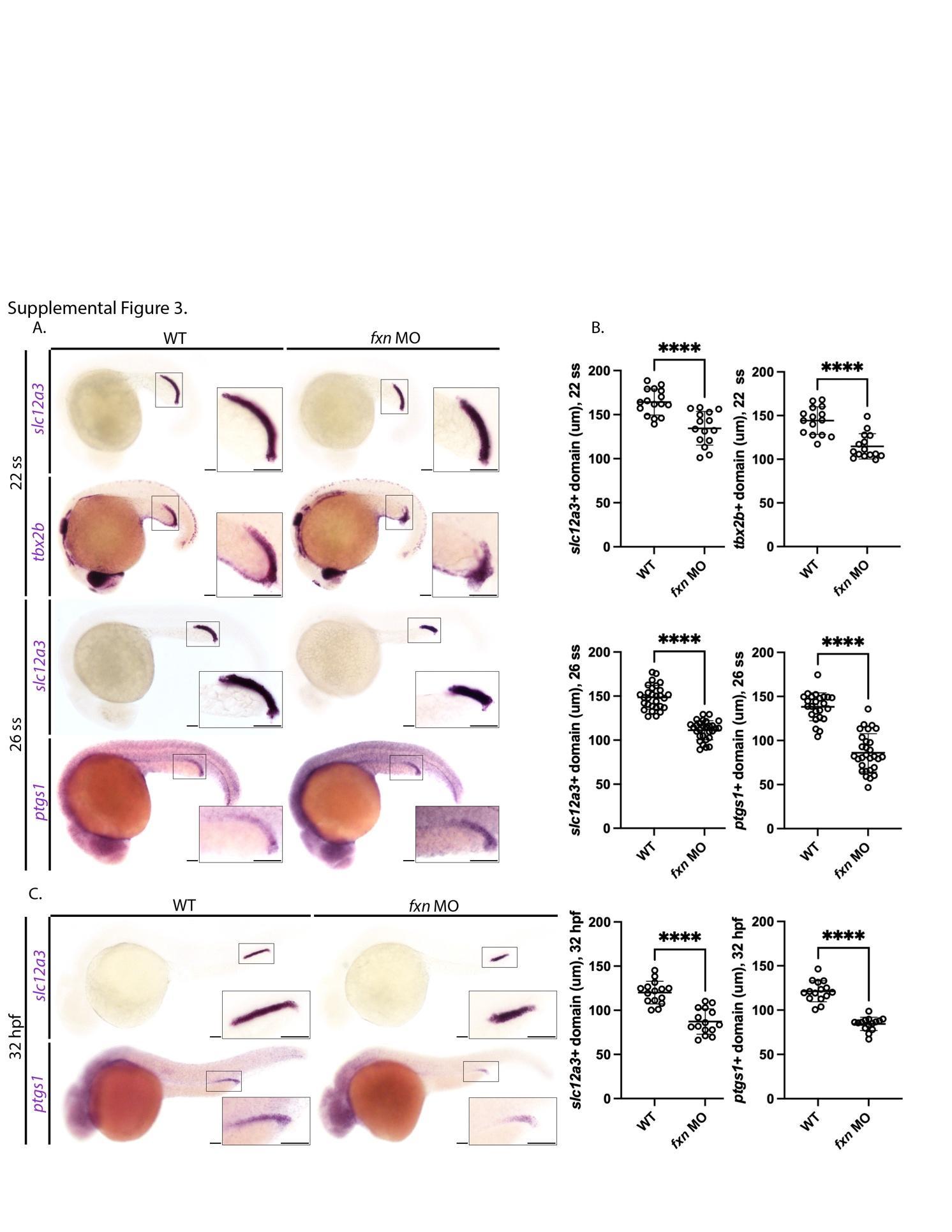
**Supplementary Figure 3.** WISH reveals reduced DL length at 22 ss, 26 ss, and 32 hpf. A) The experiments at the 22 ss utilized probes *slc12a3* and *tbx2b*. The experiments at the 26 ss utilized the probes *slc12a3* and *ptgs1*. The probe *tbx2b* was used at the 22 ss instead of *ptgs1* because the latter is not expressed at 22 ss. Scale bars = 50 um. B) Unpaired t-test of the corresponding WISH experiments in panel A. C) The WISH experiments at the 32 hpf time point utilized the probes *slc12a3* and *ptgs1*. Scale bars = 50 um.  D) Unpaired t-test of the corresponding WISH experiments in panel C.


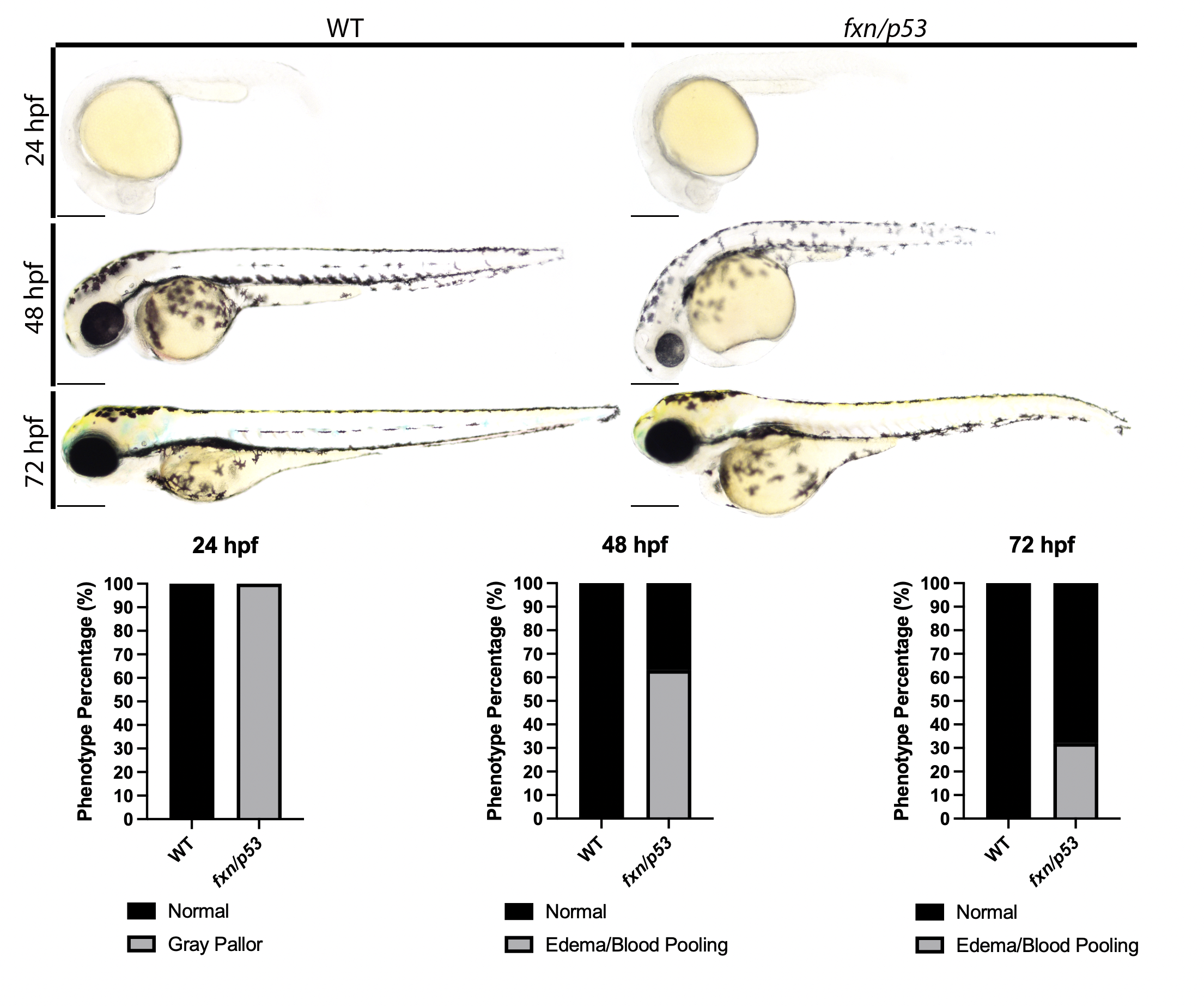


**Supplemental Figure 4.** Co-injecting a *p53* morpholino with a *fxn* morpholino does not rescue gray pallor or edema phenotypes. The gray pallor phenotype occurred in similar proportions in the *p53/fxn* animals as it did in *fxn* animals. This pattern holds true for the edema/blood pooling phenotype at both 48 and 72 hpf. Scale bars = 200 um.


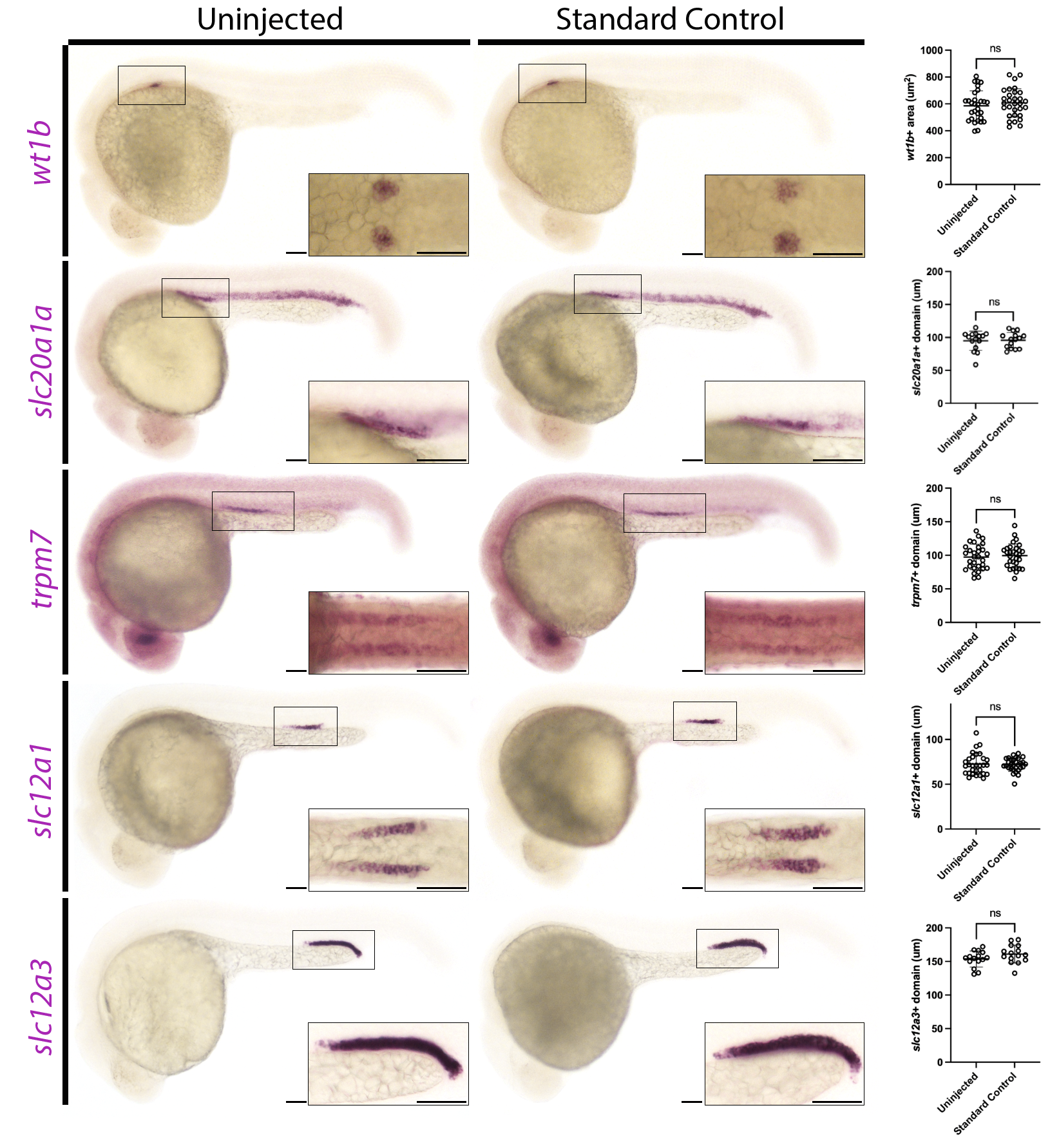


**Supplemental Figure 5.** Injecting zebrafish with a standard control morpholino does not alter the morphology of renal structures. Approximately three nanoliters of a 133 uM morpholino solution was injected at the single cell stage. Scale Bars = 50 um.


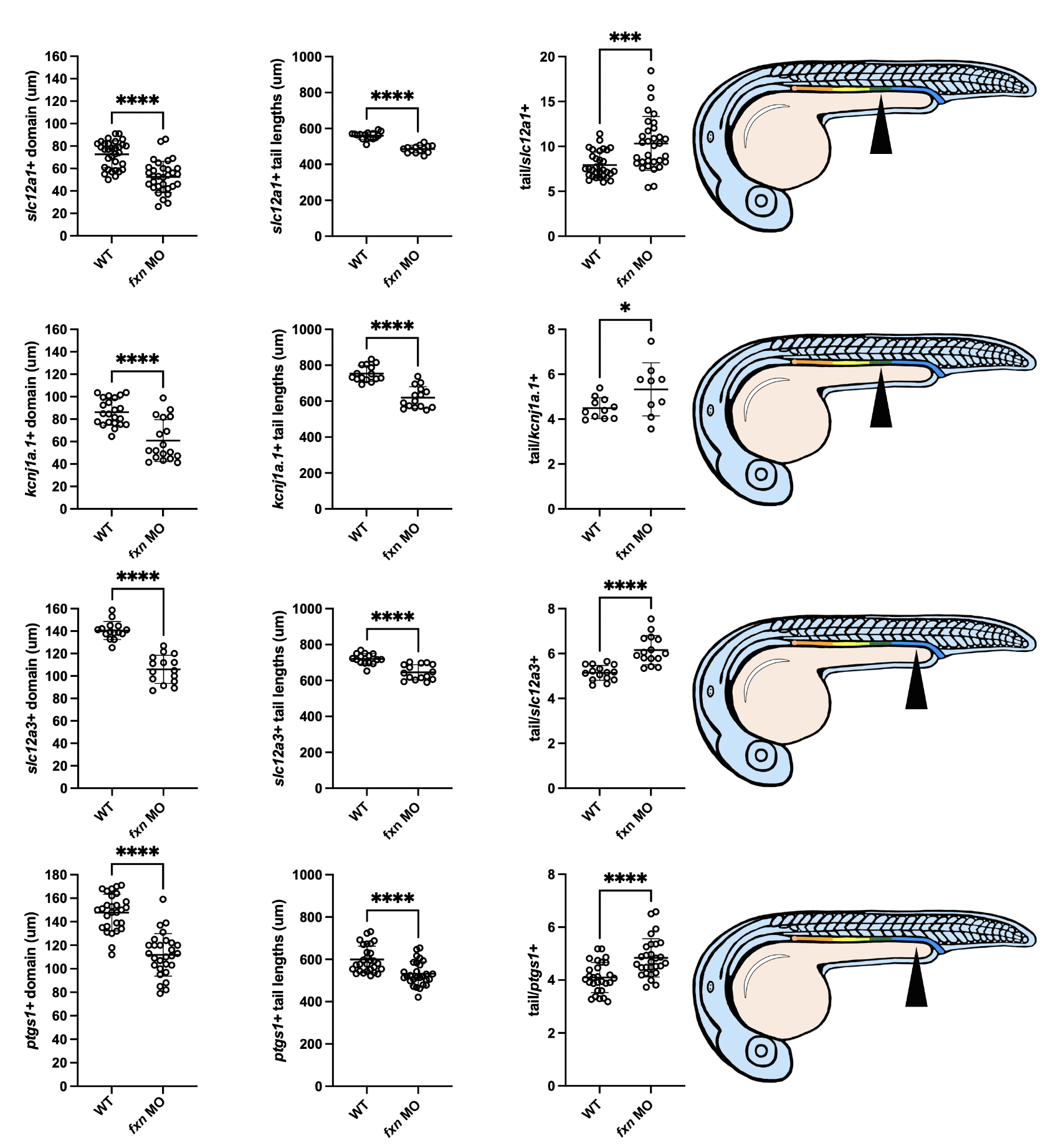


**Supplemental Figure 6.** The pronephros is negatively impacted by *fxn* deficiency to a greater magnitude than growth of the animal. *slc12a1+* domain decrease 27.8% and tails 12.8%. *kcnj1a.1+* domain decrease 27.3% and tails 17.8%. *slc12a3+* domain decrease 24.6% and tails 10.4%. *ptgs1+* domain decrease 24.3% and tails 11.2%. *cdh17+* domain decrease 9.7% and tails 12.3%. All domains % decrease 19% and tails 12.3%.
